# The Impact of Postoperative Complications After Colon Cancer Surgery on Locoregional Recurrence: A Population-Based Dutch Cohort Study

**DOI:** 10.1245/s10434-026-19637-7

**Published:** 2026-04-22

**Authors:** Eva Rademaker, Bade C. Aktas, Rudolf van den Berg, Jan Willem T. Dekker, Ignace H. J. T. de Hingh, Niels F. M. Kok, Jurriaan B. Tuynman, Johannes H. W. de Wilt, Esther C. J. Consten, Pieter J. Tanis, Henderik L. Van Westreenen, Dutch Complex Colon Cancer Initiative, O. W. Kranenburg, A. G. J. Aalbers, P. Snaebjornsson

**Affiliations:** 1 Department of Surgery, Isala, Zwolle, The Netherlands; 2 Department of Surgical Oncology and Gastrointestinal Surgery, Erasmus MC Cancer Institute, Rotterdam, The Netherlands; 3 Department of Surgery, Reinier de Graaf Gasthuis, Delft, The Netherlands; 4 Department of Surgery, Catharina Hospital, Eindhoven, The Netherlands; 5 Department of Surgical Oncology, Netherlands Cancer Institute/Antoni van Leeuwenhoek Hospital, Amsterdam, The Netherlands; 6 Department of Surgery, Amsterdam University Medical Centers, University of Amsterdam, Cancer Centre Amsterdam, Amsterdam, The Netherlands; 7 Department of Surgery, Radboud University Medical Center, Nijmegen, The Netherlands; 8 Department of Surgery, University Medical Centre Groningen, Groningen, The Netherlands; 9 Department of Surgery, Meander Medical Centre, Amersfoort, The Netherlands

**Keywords:** Locoregional recurrence, Colon cancer, Postoperative complications, Anastomotic leakage, Overall survival

## Abstract

**Background:**

In contrast to rectal cancer, the influence of postoperative complications on locoregional recurrence (LRR) in colon cancer is understudied, with conflicting reports. This study aimed to determine the impact of postoperative complications on LRR and overall survival (OS) in colon cancer.

**Methods:**

This population-based cross-sectional cohort study was carried out in 50 Dutch hospitals. Patients who underwent resection for stage I–III colon cancer between January 2014 and December 2015 were eligible. LRR comprised any intraabdominal recurrence, including peritoneal metastases.

**Results:**

A total of 7983 patients were included with a median follow-up of 62.5 months (interquartile range 58.1–80.3). Postoperative complications occurred in 2239 (28.0%) patients and included anastomotic leakage requiring re-intervention in 394 (4.9%), any other surgical complication in 944 (11.8%), and only non-surgical complications in 901 patients (11.3%). The 5-year LRR rate was 13.5%, 8.8% and 8.8%, respectively, as compared with 6.8% in patients without complications (Fine–Gray p<0.001). Only anastomotic leakage was an independent risk factor for LRR (cause-specific hazard ratio [HR] 1.45 [95% confidence interval (CI) 1.07–1.96]). Five-year OS probability was 78.9% for patients without complications versus 71.1%, 68.5%, and 62.7% for patients with anastomotic leakage, any other surgical complication, and non-surgical complications only, respectively (log-rank p<0.001). Both surgical and non-surgical complications were an independent risk factor for worse OS (HR 1.12 [95% CI 1.00–1.26); HR 1.21 [95% CI 1.08–1.36], respectively).

**Discussion:**

This study demonstrates an increased risk of LRR after all types of postoperative complications in patients with stage I–III colon cancer, but only anastomotic leakage remained independently associated with LRR. Both surgical and non-surgical complications were associated with worse OS.

**Supplementary Information:**

The online version contains supplementary material available at 10.1245/s10434-026-19637-7.

Colon cancer is one of the most frequently diagnosed malignancies globally and poses a significant health burden.^1,2^ The mainstay of curative treatment for colon cancer is surgical resection. A substantial proportion of patients experience postoperative complications, with rates varying from 25% to 40% and encompassing both surgical and non-surgical events.^3–7^ Complications after colon cancer resection are associated with increased short-term mortality, prolonged hospital stay, and higher re-admission rates.^3,4,7–9^ Additionally, complications exert an adverse effect on overall survival (OS).^3–5,9–11^ Whether an increased risk of recurrence contributes to decreased OS in patients with colon cancer who experienced postoperative complications remains inconclusive. Studies analyzing the association between complications and locoregional recurrence (LRR) are particularly limited.^8,10,12^

It is hypothesized that postoperative complications may induce systemic inflammation and transient immunosuppression.^13^ This impaired immune state could allow residual tumor cells to escape immune surveillance and proliferate, thereby increasing the risk of recurrence.^14^ It could be hypothesized that different types of complications might have varying effects on the risk of recurrence. In particular, in LRR, surgical complications, particularly anastomotic leakage, that influence local inflammatory and immune responses in the abdominal cavity potentially play a more important role than non-surgical complications, such as pneumonia or myocardial infarction.

The aim of this study was to explore whether different types of postoperative complications were related to the occurrence of LRR based on a nationwide cohort with comprehensive long-term follow-up data. The secondary aim was to study the association between surgical and non-surgical postoperative complications with OS.

## Methods

### Study Design

This study was performed as a national collaborative research project according to a snapshot design as described in the first Dutch Snapshot Research Group publication.^15^ All patients who underwent a resection for colon cancer from January 1, 2014, until December 31, 2015, were identified from the Dutch Colorectal Audit, which is a compulsory register for all Dutch hospitals conducting oncological colorectal resections. Additionally, local surgical collaborators supervised by a consultant colorectal surgeon from the 50 participating hospitals provided information regarding presentation, perioperative factors, short-term outcomes, and long-term outcomes. These data were added in electronic case report forms from October 2021 until June 2024. After data entry was completed, an independent data manager (Medical Research Data Management, Amsterdam, the Netherlands) combined Dutch Colorectal Audit and Dutch Snapshot Research Group data. Second, all patient characteristics were pseudonymized before the final dataset was provided to the research group. The research coordinator (ER) validated data input and discussed missing values and discrepancies with a senior surgeon (PJT). The study design and manuscript preparation adhered to the STROBE guidelines.^16^ Ethical approval for this study was granted by the institutional review board of Isala hospitals (Zwolle, the Netherlands; approval number 210512). This retrospective study involved anonymized patient data and imposed no additional burden on individuals. Therefore, individual informed consent from patients was not deemed necessary.

### Patient Selection and Stratification

All patients aged ≥18 years who underwent curatively intended oncological resection of a histologically proven adenocarcinoma of the colon (cecum to sigmoid take-off) in the years 2014 and 2015 were included. Patients with an appendiceal tumor, macroscopical positive resection margins (R2), synchronous metastases, short-term mortality within 30 days, or pT0 tumors were excluded.

Patients were stratified into four groups based on the absence or presence of different types of postoperative complications: (1) no complications; (2) anastomotic leakage, with or without additional (non-)surgical complications; (3) other surgical complications (excluding anastomotic leakage), with or without additional non-surgical complications; and (4) non-surgical complications only. Anastomotic leakage was expected to have the highest impact, so we decided to analyze this complication separately. Surgical complications were assumed to have a greater potential association than non-surgical complications with LRR. Therefore, patients with both surgical and non-surgical complications were analyzed in the surgical complication group.

Complications and re-interventions were registered during the first 30 days after resection. Anastomotic leakage was defined as clinical suspicion of leakage of intestinal fluid or abscess formation at the site of the anastomosis (including blind-ending loop(s)) requiring a re-intervention (radiological, surgical, endoscopic). Surgical complications included postoperative bleeding, prolonged ileus, fascial dehiscence, colonic perforation unrelated to the anastomosis, urinary leakage due to ureter or bladder perforation, or wound infection. Non-surgical complications consisted of pulmonary, cardiac, thrombo-embolic, neurological, and infectious complications not related to surgery.

### Outcome Measures and Definitions

The primary endpoint of this study was to assess the impact of postoperative complications on recurrence, by determining LRR and assessing independent risk factors for LRR. Secondary outcomes were 5-year OS and 5-year conditional survival (CS) after primary tumor resection. For CS analysis, only patients who were alive 1 year after initial surgery were included.

Because no universally accepted definition of LRR exists in the colon cancer literature, a broader definition was adopted to capture all recurrences arising within the abdominal compartment. The definition of LRR in this study comprised any intra-abdominal recurrence, which could be located at the anastomotic site, regional lymph node, para-iliac lymph node, abdominal wall, tumor bed, ovary, omentum, or peritoneum.^17–20^

Because of the potential association with both postoperative complications and the primary outcome measure (LRR), the presence of preoperative infectious tumor-related complications was registered. This was defined as presence of a peri-tumoral abscess, proximal diastatic perforation, tumor site perforation, or appendicitis.

### Statistical Analysis

Continuous variables are presented as mean (standard deviation) and analyzed using unpaired t-tests in cases of normal distribution. For non-normal distributed variables, outcomes are presented as median (interquartile range) and analyzed using Mann–Whitney U tests. Categorical variables are presented as count with valid percentage and analyzed using the chi-squared test or Fisher’s exact test, depending on counts per outcome. Missing data were not imputed, as each variable had less than 5% missing values. Median follow-up duration was calculated using the reverse Kaplan–Meier estimator, which accounts for censoring in survival data.

LRR was analyzed within a competing risks framework. Cumulative incidence functions were used to estimate the probability of LRR over time while treating distant metastasis and death as competing events because they preclude the subsequent occurrence of LRR. This approach avoids the overestimation of event probabilities that occurs when standard Kaplan–Meier methods are applied in the presence of competing risks.^21^ All patients contributed follow-up time from the date of surgery until the first occurrence of LRR, a competing event, or administrative end of follow-up. Importantly, in cases where locoregional recurrence occurred simultaneously with distant metastases, these events were classified as LRR and therefore treated as events of interest. Differences in cumulative incidence between complication groups were assessed using Gray’s test.

To identify independent risk factors for LRR, cause-specific hazard models were fitted. In these models, the cause-specific hazard ratio (CSHR) of LRR was estimated while treating competing events as separate outcomes rather than censoring. This approach quantifies the instantaneous risk of developing LRR among patients who are still event free at each time point. Variables for univariable analysis were selected based on clinical relevance, existing literature, and hypothesized biological mechanisms. Variables with p<0.10 in univariable analysis were entered into the multivariable model. Variance inflation factors (VIFs) were calculated to assess multicollinearity. VIF values <5 were considered to indicate no relevant multicollinearity.

A sensitivity analysis was performed using a stricter definition of LRR excluding ovarian/adnexal, omental, and peritoneal recurrences. A second sensitivity analysis was also performed in a restricted cohort excluding patients with an R1 resection to assess the robustness of the observed associations.

For OS and CS, Cox proportional hazards regression was used. Clinically relevant variables were opted for univariable analysis and included in multivariable analysis if p<0.1. The proportional hazards assumption was evaluated through visual inspection of the Schoenfeld residuals. Survival outcomes were plotted with Kaplan–Meier survival function stratified by group (no complications, anastomotic leakage, any other surgical complication, or non-surgical complications only) and compared using the log-rank test until date of event. In the absence of any event, the last date of follow-up was used for censoring.

Since adjuvant chemotherapy can be both associated with baseline oncologic risk (confounding by indication) and reduced as a consequence of complications (potential mediator), adjuvant chemotherapy was included as an adjustment variable in the multivariable models. This approach estimates the association between postoperative complications and LRR conditional on whether or not receiving adjuvant therapy, rather than the total effect of complications. In a subgroup analysis, adjuvant chemotherapy was excluded to ensure this covariate would not bias the estimated association as a mediator in the multivariable model.

A two-sided p-value <0.05 was considered statistically significant. All analyses were performed using R version 4.3.2 (R Group for Statistical Computing) with packages “survival” and “cmprsk”.

## Results

### Study Population

Of the 9523 patients who underwent colon cancer resection in the study period, 7983 met the inclusion criteria and were included for analysis (Supplementary Digital Content Fig. 1).

**Fig. 1:**
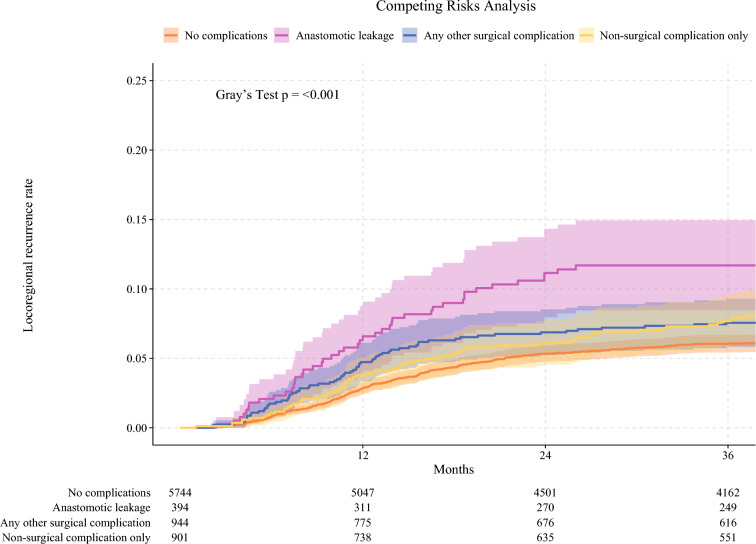
Cumulative incidence of locoregional recurrence, stratified by complication group

Patients had a median age of 70.7 years (interquartile range 65.5–77.7), and 52% were male. Data on ethnicity distribution were not available. Anastomotic leakage requiring a re-intervention was detected in 394 patients (4.9%). Any other surgical complication was detected in 944 patients (11.8%). In the group of patients with any other surgical complication, 276 (29.2%) required a re-intervention. In total, non-surgical complications occurred in 1486 patients (18.6%). Of these, 901 (60.6%) had non-surgical complications only.

Table 1 shows the baseline characteristics for each of the four patient groups: no complications, anastomotic leakage, any other surgical complication, and non-surgical complications only. Patients who experienced postoperative complications were older, had higher American Society of Anesthesiologists (ASA) scores, and primary presentation was more often symptomatic (including anemia, obstruction, or with an pre-operative infectious tumor-related complication). Surgery was more often in an acute or urgent setting, and the surgical approach more often converted to open or primary open. Patients with any complication more often received neoadjuvant therapy. Differences in surgical details and pathological parameters were also observed. Patients with complications had relatively more often undergone multivisceral resection and had higher proportions of more advanced T categories, and resection margins were more often compromised (R1) or not reported (Rx) in the three groups with complications.

**Table 1:** Baseline characteristics

Characteristic	Overall N = 7983	No complications N = 5744	Anastomotic leakage N = 394	Any other surgical complication N = 944	Non-surgical complication only N = 901	p-Value
Age at resection	70.7 (65.5–77.7)	69.8 (64.8–76.6)	69.9 (65.0–76.4)	73.7 (66.5–79.4)	75.3 (67.9–81.2)	<0.001
Sex						<0.001
Male	4154 (51.9)	2866 (49.9)	242 (61.4)	570 (60.4)	476 (52.8)	
Female	3829 (48.0)	2878 (50.1)	152 (38.6)	374 (39.6)	425 (47.2)	
ASA score						<0.001
1–2	6149 (77.1)	4648 (81.0)	285 (72.3)	633 (67.1)	583 (64.7)	
3+	1829 (22.9)	1091 (19.0)	109 (27.7)	311 (32.9)	318 (35.3)	
Missing	5	5	–	-	–	
BMI						<0.001
18.5–24.9	3002 (38.3)	2230 (39.5)	136 (35.1)	320 (34.3)	316 (35.9)	
<18.5	115 (1.5)	77 (1.4)	10 (2.6)	9 (1.0)	19 (2.2)	
25–30	3179 (40.5)	2283 (40.4)	158 (40.7)	384 (41.2)	354 (40.2)	
≥30	1551 (19.8)	1055 (18.7)	84 (21.6)	220 (23.6)	192 (21.8)	
Missing	136	99	6	11	20	
Tumor site						<0.001
Cecum	1435 (18.0)	1011 (17.6)	55 (14.0)	185 (19.6)	184 (20.4)	
Ascending colon	1381 (17.3)	993 (17.3)	52 (13.2)	167 (17.7)	169 (18.8)	
Hepatic flexure	509 (6.4)	331 (5.8)	29 (7.4)	85 (9.0)	64 (7.1)	
Transverse colon	611 (7.7)	390 (6.8)	33 (8.4)	103 (10.9)	85 (9.4)	
Splenic flexure	272 (3.4)	167 (2.9)	27 (6.9)	40 (4.2)	38 (4.2)	
Descending colon	497 (6.2)	340 (5.9)	40 (10.2)	63 (6.7)	54 (6.0)	
Sigmoid	3278 (41.1)	2512 (43.7)	158 (40.1)	301 (31.9)	307 (34.1)	
Obstruction	792 (9.9)	476 (8.3)	43 (10.9)	132 (14.0)	141 (15.6)	<0.001
Pre-operative infectious tumor complication						<0.001
No	7682 (96)	5574 (97)	364 (92)	885 (94)	859 (95)	
Iatrogenic perforation	35 (0.4)	22 (0.4)	3 (0.8)	5 (0.5)	5 (0.6)	
Clinical	266 (3.3)	148 (2.6)	27 (6.9)	54 (5.7)	37 (4.1)	
Anemia	2157 (27.1)	1413 (24.6)	126 (32.1)	332 (35.2)	286 (31.8)	<0.001
Missing	12	8	1	1	2	
Surgical setting						<0.001
Elective	7181 (90.0)	5284 (92.0)	338 (85.9)	802 (85.0)	757 (84.0)	
Acute (within 24 h)	465 (5.8)	259 (4.5)	35 (8.9)	88 (9.3)	83 (9.2)	
Urgent (within 2–7 days)	337 (4.2)	201 (3.5)	21 (5.3)	54 (5.7)	61 (6.8)	
Surgical approach						<0.001
Laparoscopic or robot-assisted	5153 (64.7)	4052 (70.7)	203 (51.7)	451 (47.9)	447 (49.7)	
Converted to open	683 (8.6)	417 (7.3)	46 (11.7)	135 (14.3)	85 (9.5)	
Primary open	2128 (26.7)	1261 (22.0)	144 (36.6)	356 (37.8)	367 (40.8)	
Missing	19	14	1	2	2	
Neoadjuvant therapy	84 (1.1)	48 (0.8)	9 (2.3)	12 (1.3)	15 (1.7)	0.01
Missing	2	2	-	-	-	
Adjuvant chemotherapy	2167 (27.3)	1662 (29.1)	82 (21.0)	230 (24.4)	193 (21.6)	<0.001
Missing	47	34	3	2	8	
Surgical procedure						<0.001
(Ext) right hemicolectomy	3526 (44.2)	2468 (43.0)	138 (35.0)	469 (49.7)	451 (50.1)	
Transverse colectomy	161 (2.0)	106 (1.9)	10 (2.5)	23 (2.4)	22 (2.4)	
(Ext) left hemicolectomy	897 (11.2)	612 (10.7)	67 (17.0)	123 (13.0)	95 (10.5)	
Sigmoid with or without partial rectum	3122 (39.1)	2406 (41.9)	149 (37.8)	283 (30.0)	284 (31.5)	
Subtotal colectomy	175 (2.2)	91 (1.6)	23 (5.8)	31 (3.3)	30 (3.3)	
Other	102 (1.3)	61 (1.1)	7 (1.8)	15 (1.6)	19 (2.1)	
Multivisceral resection	660 (8.3)	413 (7.2)	59 (15.0)	103 (10.9)	85 (9.5)	<0.001
Missing	10	6	-	1	3	
pT-category						<0.001
(y)pT1	1028 (12.9)	820 (14.3)	42 (10.7)	83 (8.8)	83 (9.2)	
(y)pT2	1536 (19.3)	1134 (19.8)	66 (16.8)	167 (17.7)	169 (18.8)	
(y)pT3	4365 (54.7)	3095 (53.9)	208 (52.8)	560 (59.3)	502 (55.7)	
(y)pT4	1029 (13.0)	676 (11.8)	78 (19.8)	133 (14.1)	142 (15.8)	
pTx/ypT0	19 (0.2)	13 (0.2)	-	1 (0.1)	5 (0.6)	
Missing	6	6	-	-	-	
pN- category						0.88
(y)pN0	5243 (65.7)	3779 (65.8)	266 (67.5)	617 (65.4)	581 (64.4)	
(y)pN1	1863 (23.4)	1329 (23.2)	91 (23.1)	218 (23.1)	225 (25.0)	
(y)pN2	868 (10.9)	628 (10.9)	37 (9.4)	108 (11.4)	95 (10.5)	
pNx	4 (<0.1)	3 (<0.1)	-	1 (0.1)	-	
Missing	5	5	-	-	-	
Resection margins						0.02
R0	7266 (98.3)	5275 (98.6)	334 (96.8)	854 (97.9)	803 (97.7)	
R1	122 (1.7)	74 (1.4)	11 (3.2)	18 (2.1)	19 (2.3)	
Rx	595	395	49	72	79	
Histological type						0.10
Well/moderately differentiated adenocarcinoma	6366 (81.8)	4631 (82.7)	303 (78.1)	740 (80.3)	692 (79.1)	
Poorly differentiated adenocarcinoma	597 (7.7)	402 (7.2)	35 (9.0)	80 (8.7)	80 (9.1)	
Mucinous adenocarcinoma	692 (8.9)	483 (8.6)	39 (10.0)	85 (9.2)	85 (9.7)	
Signet-ring cell carcinoma	61 (0.8)	38 (0.7)	6 (1.5)	9 (1.0)	8 (0.9)	
Other histological type	65 (0.8)	43 (0.8)	5 (1.3)	7 (0.8)	10 (1.1)	
Missing	202	147	6	23	26	
Lymphovascular invasion	1539 (19.7)	1075 (19.1)	80 (20.6)	195 (21.2)	189 (21.7)	0.16
Missing	164	103	6	24	31	

Dare presented as n (%) or median (interquartile range) unless otherwise indicated.

ASA: American Society of Anesthesiologists; BMI:L body mass index.

Of the 3161 patients with an indication for adjuvant chemotherapy, 2723 had stage III disease and 427 had high-risk stage II disease. Among these, 1875 of the 2723 stage III patients (with eight missing chemotherapy status values) and 124 of the 427 stage II patients (with three missing chemotherapy status values) received adjuvant treatment. Information on the timing of chemotherapy initiation was not available in the dataset. Adjuvant chemotherapy was associated with postoperative complications: 69.1% of patients without complications received chemotherapy compared with 46.4% with anastomotic leakage, 54.5% with other surgical complications, and 47.2% with non-surgical complications (overall p<0.001).

### Locoregional Recurrence

Median follow-up after primary resection was 62.5 months (interquartile range 58.1–80.3). In total, 581/7983 (7.3%) of the patients developed an LRR during follow-up. The cumulative incidence of LRR was 3.4% after 1 year, 6.7% after 3 years and 7.6% after 5 years. The different LRR locations had the following 5-year rates using Kaplan–Meier analysis: 1.2% tumor bed, 3.9% peritoneum, 0.1% omentum, 0.4% ovary, 1.1% abdominal wall, 1.6% anastomosis, 1.1% regional lymph node, 0.7% para-iliac, and 0.2% unspecified LRR (Supplementary Digital Content Table 1).

Stratified analysis of patients without complications, and patients with either anastomotic leakage, any other surgical complication, or non-surgical complications only, revealed different LRR rates: 2.8%, 6.6%, 4.7%, and 3.7% at 1 year; 6.0%, 11.7%, 7.6%, and 7.7% at 3 years; and 6.8%, 13.5%, 8.8%, and 8.8% at 5 years, respectively (overall Fine–Gray p<0.001; Fig. 1).

Table 2 summarizes possible risk factors for the development of LRR based on 7760 patients with complete information on all covariates included in the model, of whom 570 developed LRR. In this cause-specific hazard analysis, there were no covariates with VIF >5, indicating no serious multicollinearity. After correcting for potential confounders as identified in univariable analysis, anastomotic leakage requiring reintervention was independently associated with an increased risk of developing LRR (CSHR 1.45 [95% confidence interval (CI) 1.07–1.96]). Other surgical complications and non-surgical complications only were not independently associated with LRR.

**Table 2:** Cause-specific hazard analysis for locoregional recurrence rate using competing risks within a population of patients who underwent R0–1 resection of stage I–III colon cancer and survived the first 30 days postoperatively

Variable	Univariable		Multivariable	
	CSHR (95% CI)	Significance^a^	CSHR (95% CI)	Significance
Obstruction				
No	*Ref*			
Yes	2.82 (2.31–3.43)	<0.001	1.49 (1.14–1.95)	0.003
Anemia				
No	*Ref*			
Yes	3.39 (2.88–3.99)	<0.001	1.88 (1.57–2.25)	<0.001
Surgical approach				
Laparoscopic or robot-assisted	*Ref*			
Converted to open	2.02 (1.56–2.62)	<0.001	0.86 (0.65–1.13)	0.28
Primary open	2.12 (1.78–2.53)	<0.001	0.8 (0.65–1.00)	0.05
Emergency setting				
Elective	*Ref*			
Acute	3.04 (2.39–3.87)	<0.001	1.31 (0.94–1.84)	0.11
Urgent	2.32 (1.71–3.16)	<0.001	1.1 (0.77–1.59)	0.60
Multivisceral resection				
No	*Ref*			
Yes	4.53 (3.77–5.45)	<0.001	0.78 (0.61–0.98)	0.03
Tumor sidedness				
Left	*Ref*			
Right	1.33 (1.13–1.57)	<0.001	1.65 (1.38–1.99)	<0.001
pT stage				
(y)pT0-2,x	*Ref*			
(y)pT3	6.86 (4.60–10.25)	<0.001	3.32 (2.20–5.01)	<0.001
(y)pT4	31.35 (20.94–46.92)	<0.001	6.89 (4.45–10.67)	<0.001
pN stage				
(y)pN0-x	*Ref*			
(y)pN1	2.71 (2.22–3.30)	<0.001	2.02 (1.60–2.54)	<0.001
(y)pN2	6.58 (5.40–8.03)	<0.001	3.41 (2.64–4.39)	<0.001
Preoperative infectious tumor complication				
No	*Ref*			
Iatrogenic or clinical preoperative infectious tumor complication	4.05 (3.16–5.21)	<0.001	1.1 (0.81–1.48)	0.54
Resection margins				
R0	*Ref*			
R1	10.43 (7.58–14.34)	<0.001	4.29 (3.02–6.10)	<0.001
Rx	10.91 (9.18–12.96)	<0.001	6.1 (4.92–7.55)	<0.001
Neoadjuvant therapy				
No	*Ref*			
Yes	1.74 (0.93–3.26)	0.08	1.36 (0.69–2.68)	0.37
Histological type				
Well to moderately differentiated adenocarcinoma	*Ref*			
Other	2.01 (1.69–2.40)	<0.001	1.28 (1.07–1.53)	0.01
Lymphovascular invasion				
No	*Ref*			
Yes	4.54 (3.86–5.35)	<0.001	2.03 (1.69–2.44)	<0.001
Adjuvant chemotherapy				
No	*Ref*			
Yes	2.09 (1.77–2.46)	<0.001	0.65 (0.54–0.80)	<0.001
Postoperative complications				
No	*Ref*			
Anastomotic leakage	2.21 (1.66–2.96)	<0.001	1.45 (1.07–1.96)	0.02
Any other surgical complication	1.40 (1.10–1.78)	0.01	1.14 (0.89–1.47)	0.30
Non-surgical complication only	1.35 (1.05–1.74)	0.02	0.96 (0.74–1.24)	0.75

CI, confidence interval; CSHR, cause-specific hazard ratio.

^a^Included in multivariable analysis in case p<0.1

Other factors associated with LRR were presentation with obstruction, presentation with anemia, acute resection, depth of tumor invasion (both (y)pT3 and (y)pT4), presence of positive lymph nodes (both (y)pN1 and (y)pN2), positive or unknown resection margins, adenocarcinoma with poor differentiation, and lymphovascular invasion. Factors associated with a decreased risk of LRR were multivisceral resection, primary open procedure, and administration of adjuvant chemotherapy.

The sensitivity analysis where ovarian/adnexal, omental, and peritoneal recurrences were excluded showed similar trends. In the multivariable cause-specific hazard analysis, anastomotic leakage requiring reintervention was independently associated with an increased risk of developing LRR (CSHR 1.70 [95% CI 1.09–2.63]). Other surgical complications and non-surgical complications only were again not independently associated with LRR.

In the second sensitivity analysis where R1 resections were excluded, similar trends were observed. Five-year LRR rates were 6.4% in patients without complications, 13.3% in patients with anastomotic leakage, 8.1% in patients with any other surgical complication, and 8.2% in patients with non-surgical complications only (overall Fine–Gray p<0.001; Supplementary Digital Content Fig. 2). In multivariable cause-specific hazard analysis, anastomotic leakage requiring reintervention was still independently associated with an increased risk of developing LRR (CSHR 1.59 [95% CI 1.17–2.17]). Other surgical complications and non-surgical complications only were still not independently associated with LRR.

**Fig. 2:**
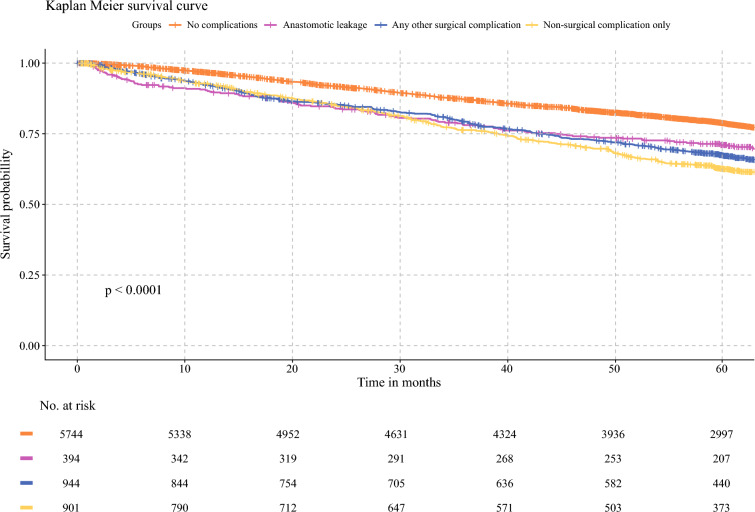
Overall survival plot, stratified per complication group

Another subgroup analysis where adjuvant chemotherapy was excluded revealed consistent observations. The only postoperative complication associated with an increased risk of LRR remained anastomotic leakage (CSHR 1.58 [95% CI 1.17–2.14]).

### Overall Survival

Five-year OS was 78.9% for patients without complications, 71.1% for patients with anastomotic leakage, 68.5% for patients with any other surgical complication, and 62.7% for patients with non-surgical complications only (log-rank p<0.001), as demonstrated in Fig. 2. The 5-year CS was 81.5%, 78.6%, 73.2%, and 67.6%, respectively (log-rank p<0.001, Supplementary digital content Fig. 3).

Multivariable Cox regression analyses were both performed for OS and CS (Table 3). For OS, any other surgical complication (HR 1.12 [95% CI 1.00–1.26]) and presence of non-surgical complications only (HR 1.21 [95% CI 1.08–1.36]) were independent predictors, but not anastomotic leakage. When patients who died in the first postoperative year were excluded, only non-surgical complications remained independently associated with CS (HR 1.20 [95% CI 1.06–1.36]).

**Table 3:** Cox regression analysis for overall survival and 1-year conditional overall survival within a population of patients who underwent R0–1 resection of stage I–III colon cancer and survived the first 30 days postoperatively, and 7135 patients who survived the first year, respectively

Variable	Univariable OS		Multivariable OS		Univariable 1-CS		Multivariable 1-CS	
	HR (95% CI)	Significance^a^	HR (95% CI)	Significance	HR (95% CI)	Significance^a^	HR (95% CI)	Significance
*Age at resection, years*								
<50	*Ref*				*Ref*		*Ref*	
50–75	1.76 (1.29–2.40)	<0.001	1.63 (1.18–2.27)	0.003	1.68 (1.22–2.33)	0.002	1.6 (1.14–2.26)	0.01
≥75	4.12 (3.02–5.61)	<0.001	2.96 (2.12–4.12)	<0.001	3.98 (2.88–5.49)	<0.001	3.04 (2.15–4.3)	<0.001
*Sex*								
Male	*Ref*				*Ref*			
Female	0.93 (0.86–1.00)	0.05	0.83 (0.77–0.90)	<0.001	0.93 (0.86–1.01)	0.09	0.84 (0.77–0.92)	<0.001
*ASA score*								
1–2	*Ref*				*Ref*			
3+	2.23 (2.07–2.41)	<0.001	1.64 (1.51–1.79)	<0.001	2.09 (1.92–2.27)	<0.001	1.56 (1.42–1.70)	<0.001
*BMI*								
18.5–24.9	*Ref*				*Ref*			
<18.5	1.86 (1.44–2.40)	<0.001	1.22 (0.94–1.58)	0.14	1.55 (1.14–2.10)	0.01	1.07 (0.78–1.46)	0.68
25-30	0.97 (0.89–1.05)	0.50	0.94 (0.86–1.02)	0.15	0.99 (0.91–1.09)	0.87	0.95 (0.87–1.04)	0.29
≥30	0.98 (0.88–1.08)	0.64	0.94 (0.85–1.05)	0.30	1.02 (0.91–1.13)	0.79	0.98 (0.88–1.10)	0.79
*Obstruction*								
No	*Ref*				*Ref*			
Yes	1.62 (1.46–1.81)	<0.001	1.20 (1.03–1.40)	0.02	1.51 (1.35–1.70)	<0.001	1.22 (1.02–1.45)	0.03
*Anemia*								
No	*Ref*				*Ref*			
Yes	1.52 (1.41–1.64)	<0.001	1.15 (1.05–1.25)	0.002	1.46 (1.35–1.59)	<0.001	1.14 (1.04–1.25)	0.01
*Surgical approach*								
Laparoscopic or robot-assisted	*Ref*				*Ref*			
Converted to open	1.30 (1.15–1.48)	<0.001	1.08 (0.94–1.23)	0.28	1.22 (1.07–1.40)	0.004	1.06 (0.92–1.22)	0.44
Primary open	1.52 (1.40–1.64)	<0.001	1.09 (0.99–1.19)	0.09	1.37 (1.26–1.50)	<0.001	1.06 (0.95–1.17)	0.30
*Emergency setting*								
Elective	*Ref*				*Ref*			
Acute	1.63 (1.42–1.86)	<0.001	1.31 (1.09–1.58)	0.004	1.43 (1.23–1.65)	<0.001	1.22 (1.00–1.5)	0.06
Urgent	1.70 (1.45–1.99)	<0.001	1.18 (0.97–1.44)	0.09	1.57 (1.32–1.87)	<0.001	1.12 (0.9–1.39)	0.31
*Multivisceral resection*								
No	*Ref*				*Ref*			
Yes	1.05 (0.92–1.18)	0.47			0.91 (0.80–1.05)	0.19		
*Tumor side*								
Left	*Ref*				*Ref*			
Right	1.28 (1.19–1.38)	<0.001	1.1 (1.01–1.19)	0.04	1.21 (1.12–1.31)	<0.001	1.06 (0.97–1.16)	0.23
pT-stage								
(y)pT0-2,x	*Ref*				*Ref*			
(y)pT3	1.28 (1.17–1.40)	<0.001	1.02 (0.92–1.13)	0.68	1.23 (1.12–1.35)	<0.001	1.02 (0.92–1.14)	0.67
(y)pT4	2.19 (1.96–2.45)	<0.001	1.44 (1.26–1.65)	<0.001	1.91 (1.69–2.16)	<0.001	1.37 (1.18–1.59)	<0.001
*pN stage*								
(y)pN0-x	*Ref*				*Ref*			
(y)pN1	1.20 (1.10–1.32)	<0.001	1.49 (1.33–1.66)	<0.001	1.20 (1.09–1.32)	<0.001	1.5 (1.33–1.68)	<0.001
(y)pN2	2.03 (1.83–2.25)	<0.001	2.40 (2.10–2.74)	<0.001	1.79 (1.59–2.01)	<0.001	2.19 (1.89–2.55)	<0.001
*Preoperative infectious tumor complication*								
No	*Ref*				*Ref*			
Iatrogenic perforation	0.72 (0.41–1.27)	0.25			0.78 (0.44–1.38)	0.39		
Clinical	1.13 (0.95–1.36)	0.17			0.95 (0.77–1.17)	0.63		
*Resection margins*								
R0	*Ref*				*Ref*			
R1	1.23 (0.96–1.56)	0.10	1.08 (0.83–1.42)	0.55	0.92 (0.69–1.23)	0.58	0.91 (0.66–1.27)	0.59
Rx	1.56 (1.39–1.76)	<0.001	1.26 (1.11–1.44)	<0.001	1.58 (1.39–1.78)	<0.001	1.38 (1.20–1.59)	<0.001
Neoadjuvant therapy								
No	*Ref*				*Ref*			
Yes	0.56 (0.38–0.82)	0.003	0.66 (0.44–0.98)	0.04	0.52 (0.34–0.78)	0.002	0.64 (0.41–0.98)	0.04
*Histological type*								
Well/moderately differentiated adenocarcinoma	*Ref*				*Ref*			
Poorly differentiated adenocarcinoma	1.50 (1.32–1.71)	<0.001	1.19 (1.04–1.37)	0.01	1.25 (1.08–1.45)	0.003	1.07 (0.92–1.25)	0.38
Mucinous adenocarcinoma	1.20 (1.06–1.35)	0.004	1.16 (1.02–1.31)	0.03	1.17 (1.03–1.34)	0.02	1.16 (1.01–1.33)	0.03
Signet cell carcinoma	1.88 (1.36–2.62)	<0.001	1.34 (0.95–1.89)	0.09	1.70 (1.18–2.46)	0.004	1.28 (0.87–1.88)	0.21
Other	1.82 (1.29–2.56)	<0.001	1.57 (1.10–2.26)	0.01	1.49 (1.00–2.23)	0.05	1.40 (0.91–2.15)	0.12
*Lymphovascular invasion*								
No	*Ref*				*Ref*			
Yes	1.72 (1.58–1.87)	<0.001	1.42 (1.29–1.56)	<0.001	1.56 (1.42–1.71)	<0.001	1.35 (1.22–1.50)	<0.001
*Adjuvant chemotherapy*								
No	*Ref*				*Ref*			
Yes	0.76 (0.70–0.83)	<0.001	0.54 (0.48–0.61)	<0.001	0.77 (0.70–0.85)	<0.001	0.57 (0.50–0.65)	<0.001
*Postoperative complications*								
No	*Ref*				*Ref*			
Anastomotic leakage	1.23 (1.05–1.44)	0.01	1.05 (0.89–1.24)	0.58	1.04 (0.87–1.25)	0.65	0.91 (0.76–1.10)	0.34
Any other surgical complication	1.42 (1.28–1.58)	<0.001	1.12 (1.00–1.26)	0.04	1.30 (1.16–1.46)	<0.001	1.07 (0.94–1.21)	0.30
Non-surgical complication only	1.63 (1.46–1.81)	<0.001	1.21 (1.08–1.36)	<0.001	1.55 (1.38–1.74)	<0.001	1.20 (1.06–1.36)	0.004

ASA, American Society of Anesthesiologists; CI, confidence interval; HR, hazard ratio; OS, overall survival.

^a^Included in multivariable analysis in case p<0.1

## Discussion

This Dutch population-based cross-sectional cohort of 7983 patients included detailed data on different types of postoperative complications, potentially relevant confounding variables, and long-term occurrence of any form of intra-abdominal recurrence. This enabled a comprehensive analysis of the oncological risks associated with postoperative complications with appropriate statistical correction for confounding and competing risks. After 5-year follow-up, the cumulative incidence of LRR was 6.8% in case with an uncomplicated course and was higher for patients with anastomotic leakage (13.5%), other surgical complications (8.8%), and non-surgical complications only (8.8%). In multivariable analysis, only anastomotic leakage was an independent predictor for LRR. In contrast, other surgical complications than anastomotic leakage and non-surgical complications in the absence of surgical complications had an independent negative impact on OS.

In the current study, patients who experienced either anastomotic leakage, any other surgical complication, or non-surgical complications only had higher cumulative incidences of LRR than did those without complications. This observation supports the hypothesis that complications affect inflammation and immune response, potentially increasing the risk of recurrence.^13^ This hypothesis was supported by studies in an animal model, where preventing perioperative immunosuppression reduced metastatic spread and improved recurrence-free survival in murine models undergoing tumor resection.^14^ In particular, it is hypothesized that the immunological response may become exhausted following re-interventions, which is often required after leakage of a colonic anastomosis. More specifically, extra-luminal implantation of residual tumor cells due to anastomotic leakage have been a supposed trigger for both LRR and distant recurrence.^13,22–24^ The occurrence of postoperative complications may also lead to delays in the initiation of adjuvant chemotherapy or result in its complete omission, which can also contribute to impaired oncological outcomes.^25,26^

Several other factors, such as obstruction, anemia, emergency surgery, surgical approach, tumor sidedness, (y)pTN, poor differentiation, lymphovascular invasion, and positive or unreported resection margins, appeared to be associated with the risk of LRR. After correction for these well-known factors, anastomotic leakage requiring reintervention remained independently associated with LRR, whereas other surgical complications and non-surgical complications did not.

Although the relationship between anastomotic leakage and LRR has not been extensively investigated for colon cancer specifically, numerous studies in colorectal and rectal cancer have shown higher cumulative incidences of LRR after anastomotic leakage.^27–31^ For colon cancer specifically, one study suggested that anastomotic leakage was associated with a higher cumulative incidence of LRR^32^, whereas others did not report such an association.^8,11,33–36^ This might be partly explained by differences in definitions of anastomotic leakage and LRR, as well as the completeness of follow-up and rigorousness of registration of complications and recurrences. Because definitions of LRR vary across studies, the current findings should be interpreted while keeping our broader definition in mind (including peritoneal and other intra-abdominal sites). However, more importantly, the number of events, both for anastomotic leakage and LRR, was often substantially lower in the published series than in the present cohort, with the exception of one consecutive Danish series with comparable numbers.^33^ Finally, the chosen statistical method might have influenced outcomes by either taking competing risks into account or not.

Data on LRR in the context of postoperative complications are extremely scarce, particularly for colon cancer, which has primarily been studied in combination with rectal cancer.^37–42^ However, a negative effect of complications on colon cancer recurrence has been reported in selected cohorts.^10,12^ Klaver et al.^12^ primarily focused on intra-abdominal recurrence rates after T4 colon cancer in relation to surgical site infections (SSI) and reported a higher rate of 5-year intra-abdominal recurrence for these patients. Furthermore, Cienfuegos et al.^10^ reported a significant difference between local recurrences in patients with and without complications (overall rate 7.8% and 2.4%, respectively).

The association between OS and complications was investigated by Warps et al.^5^ using a comparable population-based dataset. Their findings indicated that non-surgical complications had a greater impact on survival than surgical complications, which is in line with our findings. As also noted by Warps et al.^5^, the question remains as to whether non-surgical complications themselves drive the observed reduction in survival or whether they predominantly occur in an already more vulnerable subgroup, characterized in the current study by older age, higher ASA scores, more acute and open procedures, and a greater incidence of obstruction, which may not be fully accounted for despite multivariable adjustment.

In this study, 28.0% of patients experienced a postoperative complication within 30 days, which is comparable to rates in the reported data.^3,4,6,7^ However, comparability in the literature is limited, and reported incidences vary widely because of the diverse definitions and classifications used to describe postoperative outcomes. The influence of these varying definitions is described in a meta-analysis that examined postoperative complications in colorectal surgery, categorizing them as anastomotic leakage (defined according to each individual study included) and SSI based on guidelines from the Centers for Disease Control and Prevention.^28^ Their broad inclusion of extra-abdominal infections under SSI led to a less specific classification. Similar issues arise with the use of the Clavien–Dindo score^6,10,12,43,44^ and in studies focusing solely on SSI.^39,40,45^ Moreover, intra-abdominal infections are also classified inconsistently. For example, Sanchez et al.^46^ considered only anastomotic leakage and intra-abdominal abscesses, whereas Sueda et al.^47^ extended the definition to include a positive drainage culture. These discrepancies highlight the need for standardized definitions and classifications to ensure that both incidence rates and potential long-term outcomes of postoperative complications can be accurately compared.

An inherent but significant limitation of our study is its retrospective design, which is inherently susceptible to various types of bias, including information bias and selection bias. Although data were collected across 50 hospitals, the diversity in clinical practices and the use of different (diagnostic) protocols across institutions may introduce heterogeneity in the data, potentially affecting the results. Additionally, certain data might be inadequately documented, for example because of the limited extent of disease, which could lead to systematic underreporting of long-term specific outcomes or complications.

Despite efforts to minimize these biases by validating the data through overlap with national registries and direct queries to local collaborators, the possibility remains that some data could be incomplete or inaccurate.

Another limitation of our study is that it captured and analyzed complications reported within the initial 30 days after colon cancer surgery, but complications might occur beyond this period. These potential late-onset complications were not recorded in the dataset for all included patients, which may have led to an underestimation of the true incidence of late-onset complications. Moreover, the dataset lacked information on the exact timing of adjuvant chemotherapy, which is known to influence survival outcomes. Postoperative complications may delay the initiation of chemotherapy and thereby potentially affect OS. Although we adjusted for receipt of adjuvant chemotherapy in the multivariable analyses, we could not account for treatment timing. However, subgroup analyses with and without adjuvant chemotherapy as a covariate yielded comparable effect estimates, and anastomotic leakage consistently remained an independent predictor.

Furthermore, although this study is based on a nationwide Dutch cohort with high-quality audit data, surgical practices, perioperative management, and adjuvant therapy protocols may differ internationally. These differences may influence external validity, and caution is warranted when generalizing our findings to other healthcare systems.

Lastly, despite multivariable adjustment, residual confounding cannot be fully excluded. Patients who developed postoperative complications differed substantially at baseline: they were older, had higher ASA scores, more often presented with symptomatic disease, and underwent more complex procedures (including primary open surgery, urgent or emergency operations, and multivisceral resections). These inherent differences may have contributed to their increased susceptibility to postoperative morbidity and may not be fully captured by statistical adjustment.

In conclusion, this study demonstrated that anastomotic leakage after primary tumor resection was an independent risk factor for LRR alongside multiple other tumor-related, surgery-related, and histological risk factors. Moreover, postoperative other surgical and non-surgical complications adversely influenced OS, whereas this was not found for anastomotic leakage.

## Supplementary Information

Below is the link to the electronic supplementary material.

Supplementary file1 (DOCX 333 KB)

## Data Availability

Deidentified participant data and a data dictionary will be made available upon reasonable request, following publication of related manuscripts. Access requires approval of a research proposal and a signed data access agreement reviewed by the Dutch Snapshot Research Group steering committee (ER, ECJC, HLvW, PJT).
