# Supplementary material for: The Impact of Postoperative Complications After Colon Cancer Surgery on Locoregional Recurrence: A Population-Based Dutch Cohort Study
Source: Ann Surg Oncol. 2026 Apr 22;33(8):6972–88. doi: 10.1245/s10434-026-19637-7 (PMC13337792; doi:10.1245/s10434-026-19637-7)
Supplement: Supplementary file 1 — Supplementary file1 (DOCX 333 KB) [file 10434_2026_19637_MOESM1_ESM.docx]

## List of supplemental digital content

1. **Figure 1**: Flowchart inclusion and exclusion criteria
2. **Table 1**: Five-year locoregional recurrence rate per subtype, stratified by complication group
3. **Figure 2**: Cumulative incidence of locoregional recurrence, stratified by complication group in absence of R1 resections
4. **Figure 3**: One year conditional overall survival plot, stratified per complication group

**Supplementary digital content Figure 1**: Flowchart inclusion and exclusion criteria

Anastomotic leakage
n=394 (4.9%)

Snapshot colon cancer cohort
n=9,523

Included patients
n=7,983

No complications
n=5,744 (72.0%)

Any other surgical complication
n=944 (11.8%)

Non-surgical complication only
n=901 (11.3%)

Appendiceal tumor (n=74)
R2-resection (n=63)
Synchronous metastases (n=1,205)
Unknown synchronous metastases (n=19)
Mortality ≤30 days (n=149)
pT0 (n=30)

**Supplementary digital content Table 1**: Five-year locoregional recurrence rate per subtype, stratified by complication group

|  | **Overall**  N = 7,983 | **No complications**  N = 5,744 | **Anastomotic leakage**  N = 394 | **Any other surgical complication**  N = 944 | **Non-surgical complication only**  N = 901 |
| --- | --- | --- | --- | --- | --- |
| Tumor bed recurrence | 1.2% | 1.1% | 1.6% | 2.2% | 1.1% |
| Peritoneal recurrence | 3.9% | 3.3% | 6.7% | 5.5% | 5.1% |
| Omental recurrence | 0.1% | 0.1% | - | 0.5% | - |
| Ovarian/adnexal recurrence | 0.4% | 0.4% | 0.7% | 0.4% | 0.5% |
| Abdominal wall recurrence | 1.1% | 1.0% | 2.6% | 0.8% | 1.3% |
| Anastomotic recurrence | 1.6% | 1.6% | 2.4% | 1.3% | 1.6% |
| Regional LN recurrence | 1.1% | 0.9% | 1.5% | 1.6% | 1.8% |
| Para-iliac recurrence | 0.7% | 0.7% | 1.2% | 0.3% | 1.2% |
| NOS recurrence | 0.2% | 0.1% | - | 0.6% | 0.4% |

**Supplementary digital content Figure 2**: Cumulative incidence of locoregional recurrence, stratified by complication group in absence of R1 resections

**
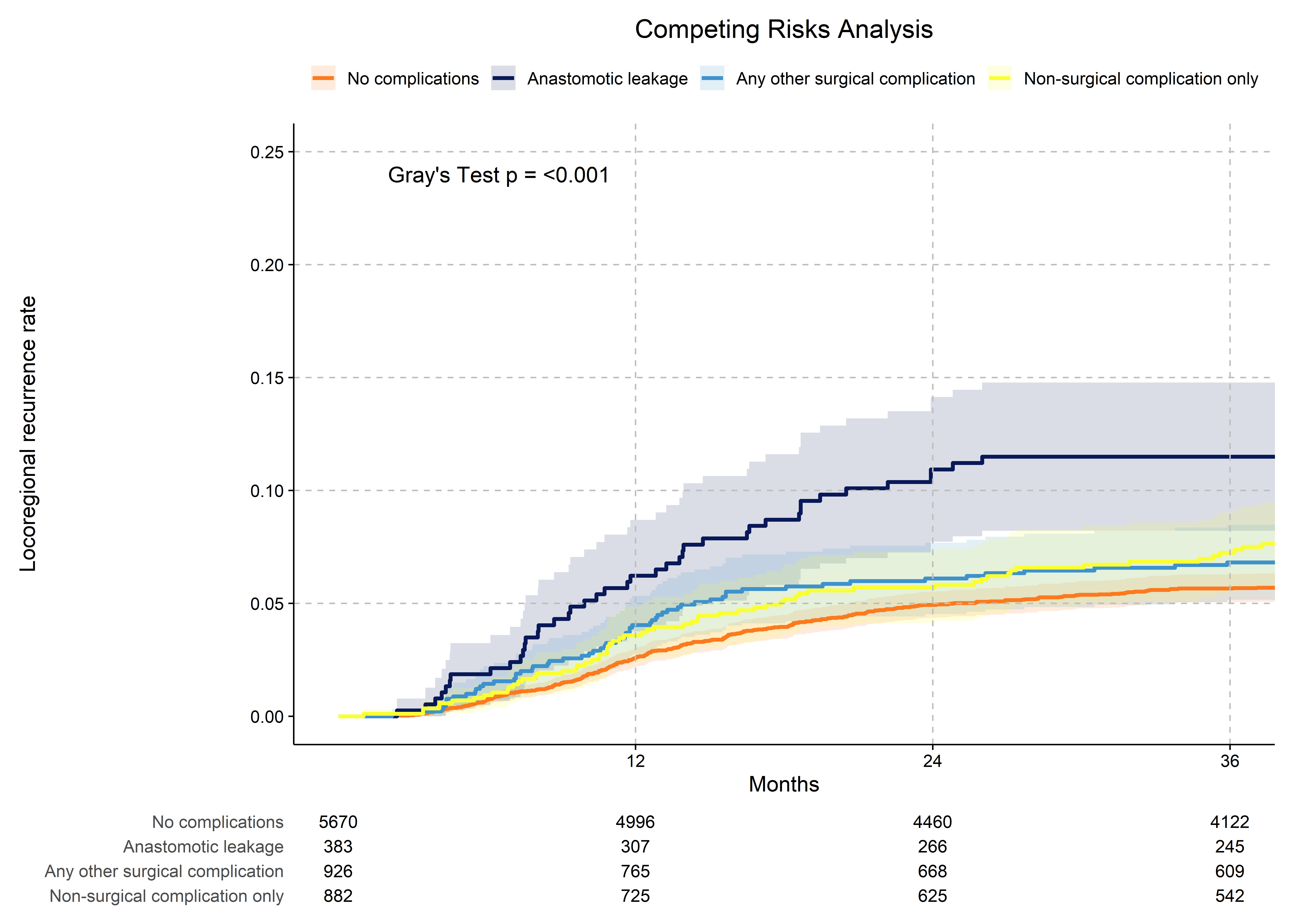
**

**Supplementary digital content Figure 3**: One year conditional overall survival plot, stratified per complication group

**
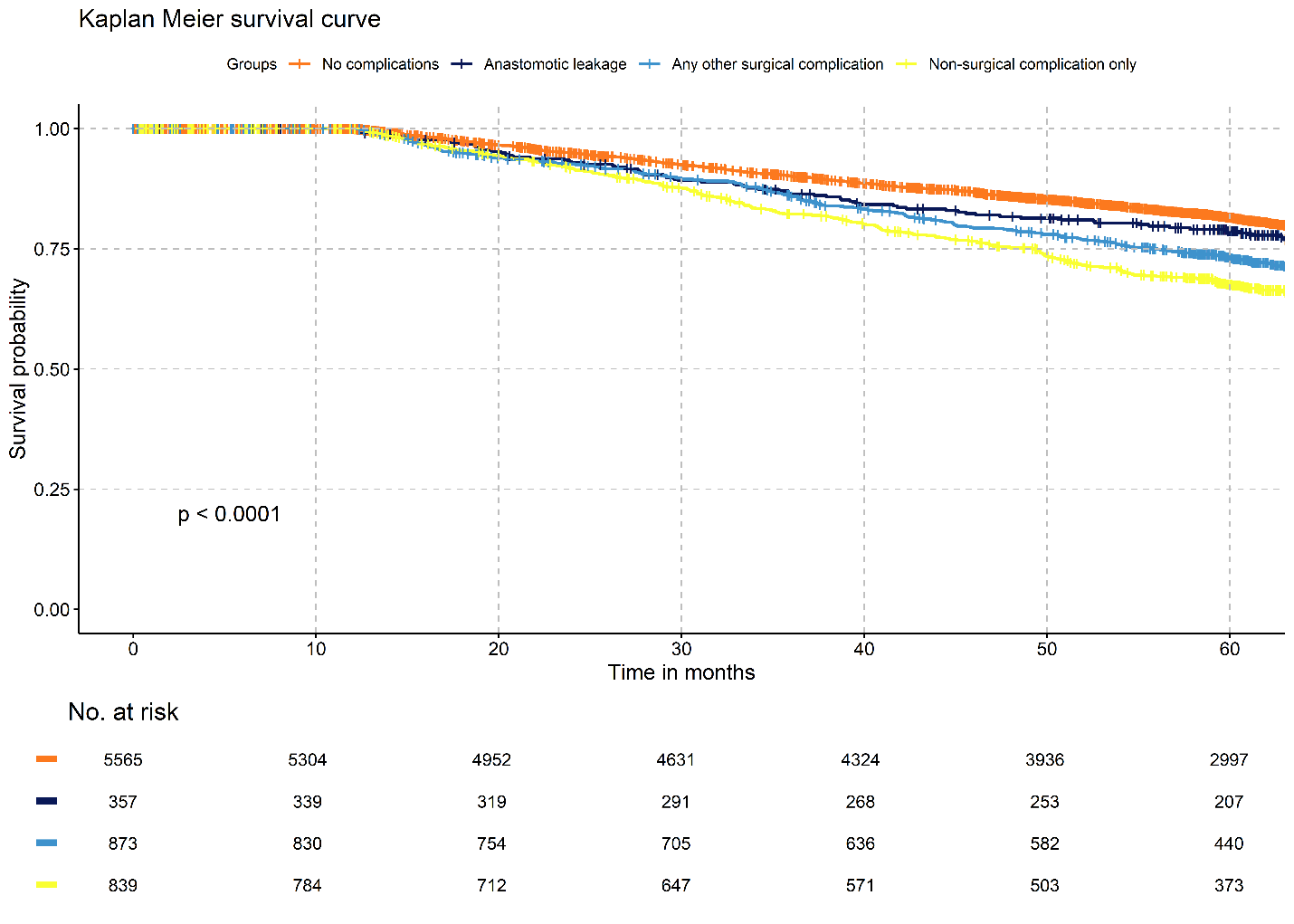
**
